# Selective activation and proliferation of a quiescent stem cell population in the neuroepithelial body microenvironment

**DOI:** 10.1186/s12931-018-0915-8

**Published:** 2018-10-26

**Authors:** Line Verckist, Isabel Pintelon, Jean-Pierre Timmermans, Inge Brouns, Dirk Adriaensen

**Affiliations:** Laboratory of Cell Biology and Histology, Department of Veterinary Sciences, University of Antwerp, Universiteitsplein 1, 2610 Wilrijk, Antwerpen Belgium

**Keywords:** Airway epithelium, Neuroepithelial body microenvironment, Stem cell niche, Clara-like cells, Pulmonary neuroendocrine cells, Lipopolysaccharide, Proliferation

## Abstract

**Background:**

The microenvironment (ME) of neuroepithelial bodies (NEBs) harbors densely innervated groups of pulmonary neuroendocrine cells that are covered by Clara-like cells (CLCs) and is believed to be important during development and for adult airway epithelial repair after severe injury. Yet, little is known about its potential stem cell characteristics in healthy postnatal lungs.

**Methods:**

Transient mild lung inflammation was induced in mice via a single low-dose intratracheal instillation of lipopolysaccharide (LPS). Bronchoalveolar lavage fluid (BALF), collected 16 h after LPS instillation, was used to challenge the NEB ME in ex vivo lung slices of control mice. Proliferating cells in the NEB ME were identified and quantified following simultaneous LPS instillation and BrdU injection.

**Results:**

The applied LPS protocol induced very mild and transient lung injury. Challenge of lung slices with BALF of LPS-treated mice resulted in selective Ca^2+^-mediated activation of CLCs in the NEB ME of control mice. Forty-eight hours after LPS challenge, a remarkably selective and significant increase in the number of divided (BrdU-labeled) cells surrounding NEBs was observed in lung sections of LPS-challenged mice. Proliferating cells were identified as CLCs.

**Conclusions:**

A highly reproducible and minimally invasive lung inflammation model was validated for inducing selective activation of a quiescent stem cell population in the NEB ME. The model creates new opportunities for unraveling the cellular mechanisms/pathways regulating silencing, activation, proliferation and differentiation of this unique postnatal airway epithelial stem cell population.

## Background

The postnatal lung is a conditionally renewing organ with a very low airway epithelial cell turnover in the absence of injury, with less than 1 % of cells dividing at any time point in several species [1, 2]. However, the lungs and airways are capable of rapidly increasing regeneration rate to replace damaged tissue, with local stem and progenitor cells re-entering the cell cycle (for reviews see [3, 4]). Adult stem cells are defined as rare cells present in different niches, with a high proliferative potential and a lifelong ability to self-renew, maintain a variety of cell populations in the steady state and/or replace damaged cells following injury [3, 5, 6].

Neuroepithelial bodies (NEBs) occur in the airway epithelium as densely innervated clusters of pulmonary neuroendocrine cells (PNECs; for review see [7]). In many species (including humans) PNECs are covered by Clara-like cells (CLCs), leaving only thin apical processes of PNECs in contact with the airway lumen. CLCs, PNECs and their extensive innervation together constitute the so-called ‘NEB microenvironment (NEB ME)’ [8–11]. CLCs have also been referred to as variant Clara cell secretory protein (CCSP)-expressing cells (vCE cells) [12].

The clusters of PNECs release bioactive substances upon stimulation [13–18] and are selectively contacted by mainly vagal afferent nerve terminals [9, 19]. Pulmonary NEBs should therefore be regarded as complex intraepithelial sensory airway receptors, capable of sensing and transducing hypoxic, mechanical, chemical and likely also other stimuli [14, 15, 20](for reviews see [9, 21–23]).

Apart from being airway sensors, NEBs may fulfill some other proposed physiological roles in the airways during fetal and perinatal life [21, 22, 24–26]. The relatively large number of NEBs encountered in the prenatal lung has been explained by their potential role in the regulation of bronchogenesis, as PNECs represent the first cell type that differentiates during embryonic lung development [27]. The possible paracrine regulation of embryonic airway epithelial cell growth by NEBs has been proposed more than 25 years ago based on cell proliferation studies, illustrating that the number of labeled divided cells progressively decreases with increasing distance from NEBs [28].

Throughout the past decade, the NEB ME has been put forward as one of the potential stem cell sources/niches that are dispersed along the epithelial lining of the mammalian respiratory tract [29–32].

The suggested stem cell capacities of the NEB ME in healthy postnatal mouse lungs were recently confirmed using an optimized laser microdissection (LMD) protocol that allows for the selective collection of high quality mRNA samples of the NEB ME [33]. Expression analysis of an extensive panel of genes, selected for their involvement in cell development, proliferation and stem cell signaling, enabled to define a stem cell ‘signature’ for the NEB ME, an indication that the NEB ME may indeed represent a functional stem cell niche in healthy postnatal mouse airways [33].

Both cell types in the NEB ME, i.e., PNECs and CLCs, have been proposed as potential airway epithelial progenitor cells [12, 34–37]. The observation that NEBs, or at least epithelial cell groups with similar characteristics, show hyperplasia in many airway diseases/disorders [38–40], and seem to play a role as precursors for small cell lung carcinoma (SCLC) [6, 34, 41], evidently suggests a role for PNECs as airway epithelial progenitors. However, the ‘stemness’ of PNECs is currently questioned since PNECs on their own were not able to restore the airway epithelium after ablation of both Clara cells (CCs) and CLCs [12]. On the other hand, self-renewing and stem cell characteristics have been assigned to CLCs/vCE cells based on lineage-tracing analysis in murine models [12, 42]. During embryonic development, cells surrounding PNECs, i.e., presumptive CLCs, remain undifferentiated [30]. CLCs/vCE cells appear to be resistant to naphthalene ablation because, unlike CCs, they do not express the cytochrome P450 2F2 isozyme [12, 43, 44]. It has been reported that CLCs in postnatal lungs show the capacity to regenerate both CCs and ciliated cells [45, 46], but that severe epithelial injury is required to activate the stem cell niche for repair [2].

Since the postnatal airway epithelium maintains a very low cell turnover at steady state, experimental damage and the follow-up of epithelial repair processes has typically been used to visualize the proliferative potential of different subsets of airway epithelial cells [1, 47]. A variety of models for severe lung injury and airway epithelial damage have been implemented to activate presumed stem cell niches and study consequent repair throughout the lung epithelium (for reviews see [3, 4, 44, 48, 49]. So far, nearly all information on the postnatal stem cell and regenerative capacities of the NEB ME, and of CLCs in particular, has also been obtained after severe experimental injury of the airway epithelium by naphthalene or genetic modification that fully ablates CCs, and from the consecutive evaluation of epithelial regeneration [12, 35, 37, 50], even in recent publications [43]. Major disadvantages of these methods, however, are the difficulty to distinguish between pathological and potentially physiological events, and the emergence of additional (undesired) hyperplasia of PNECs that apparently compromises the selectivity of activating stem cells for repair.

On the other hand, it has been well documented that all levels of severity of lung injury can be mimicked in animal models involving the application of lipopolysaccharide (LPS; intratracheal [51–53], intranasal [54], intraperitoneal [55]). In mice, intratracheal LPS instillation causes a rapid (few hours) intrapulmonary inflammatory reaction whose course is dependent of the mouse strain, LPS concentration and serotype used [52, 56, 57]. So far, however, the effects of LPS instillation on airway-associated epithelial stem cell niches have not been investigated.

Main goal of the present study was to create a minimally invasive mouse model for activation of the quiescent stem cell niche of the NEB ME. We will show that a single intratracheal instillation of a low dose of LPS –which appears to cause only a very mild and transient injury– is able to do the job. Both morphological, functional and cell proliferation assays are used to detect and quantify the relevant changes in the NEB ME that are induced by LPS challenge.

## Methods

### Animals

Lung tissue was obtained both from wild type (WT) C57BL/6 J (WT-Bl6) mice, and from a C57BL/6 J based glutamate decarboxylase 67-GFP (GAD67-GFP) mouse strain that harbors GFP fluorescent pulmonary NEBs [58] (The Jackson Laboratory, Charles River, Saint-Germain-sur-l’Arbresle, France). Both male and female mice, mainly at postnatal day (PD) 21, were used. Three-week-old mice have a much smaller lung volume –but a similar number of NEBs– compared to adults, and hence a higher density of NEBs in lung sections, resulting in more efficient studies. The results were however double-checked for adult mice (8-week-old). The young animals were housed together with their mothers in acrylic cages in an acclimatized room (12/12 h light-dark cycle; 22 ± 3 °C) and were provided with water and food ad libitum. National and international principles of laboratory animal care were followed, and experiments were approved by the local animal ethics committee of the University of Antwerp (ECD 2014–66 and 2017–49). All animals were killed by intraperitoneal (i.p.) injection of an overdose of sodium pentobarbital (Nembutal® 200 mg/kg bodyweight (BW), CEVA Sante Animale, Brussels, Belgium).

### LPS instillation

Mice were anesthetized with an i.p. injection of medetomidine (0.25 ml/kg BW; Domitor®, BE-V151742, Orion Pharma, Mechelen, Belgium) and ketamine (17.5 ml/kg BW; Nimatek®, Eurovet, Bladel, The Netherlands) to obtain a light level of surgical anesthesia, subsequently placed on an intubation table (Hallowell EMC, Pittsfield, USA) and intubated with a soft flexible cannula (24G × 3/4″; BD Angiocath; Becton Dickinson; Erembodegem, Belgium) for the intratracheal instillation of 1 mg/kg BW LPS (LPS; *Escherichia coli*; O55:B5; L6529, Sigma, Diegem, Belgium) dissolved in 50 μl of sterile saline (0.9% NaCl; Baxter, Lessen, Belgium). For evaluation of the potential effects of intratracheal instillation itself on the quantified changes in the NEB ME, sham-treated animals received an intratracheal instillation of 50 μl of sterile saline. One hundred μl of air was added to every intratracheal instillation of fluid. After completion of the instillation, anesthesia was reversed by administration of the medetomidine inhibitor atipamezole (2 ml/kg BW; Antisedan®, BE-V153352, Orion Pharma, Mechelen, Belgium) to ameliorate recovery. Animals were euthanized 16 h, 24 h, 48 h, 72 h or 7 days after challenge, as detailed further on.

### BrdU injection

As an analog of thymidine, 5-Bromo-2′-deoxyuridine (BrdU) will be incorporated in DNA during the S-phase of cell division. To mark cells that have divided during the experimental window, i.p. injections of BrdU (10 mg/kg; B5002; Sigma, Bornem, Belgium) were given immediately following intratracheal instillation in the treated groups, and at the same time point in untreated matched control mice. BrdU injections were repeated at 24 h and 48 h post-instillation. After recovery, animals were returned to their mothers until the day of euthanasia, 24 h, 48 h or 72 h after LPS challenge or any of the other treatments. Additionally, a few mice received BrdU at time points 0 h and 24 h but were sacrificed 7 days following LPS instillation. The lungs were then processed for cryosectioning and immunostaining (see further on).

### Plethysmography

Lung function parameters were measured using an unrestrained mouse Whole-Body Plethysmograph (VENT2; EMKA Technologies; Paris, France). Expiratory time (Te), relaxation time (RT), end-inspiratory pause (EIP) and tidal volume (TV) were evaluated at different time points (1 h, 2 h, 4 h, 6 h, 8 h, 12 h) after start of the experiment (LPS instillation, sham treatment, untreated controls; WT-Bl6; *n* = 4 for each group).

### Bronchoalveolar lavage

Sixteen hours after LPS or sham instillation, WT-Bl6 mice (n = 4 for each group) were euthanized and exsanguinated. Untreated WT-Bl6 mice (n = 4) served as an additional control. Bronchoalveolar lavage fluid (BALF) was collected by instillation and suction of a salt solution (2 × 1 ml; 0.9% NaCl) in the lungs via a tracheal cannula. BALF was stored at 4 °C until further use. In accordance with literature [59], the time point of 16 h after LPS treatment was chosen to allow the animals to recover completely from the anesthetics and still be able to detect effects from a combination of early and late phase mediators in the BALF. The collected BALF was first centrifuged (12 min, 150G), the cell-free supernatant fluid was removed and used as a stimulus solution for the NEB ME in lung slices in live cell imaging (LCI) experiments (see below). The cell pellet was used to prepare cytospin slides for evaluation of the cells that are present in the pulmonary air spaces. The pellet was resuspended to an end concentration of about 10^6^ cells/ml in phosphate-buffered saline (PBS; 0.01 M, pH 7.4), containing 5% bovine serum albumin (BSA; B4287, Sigma). 150 μl of this mixture was centrifuged (5 min, 800G) to generate cytospin preparations (Shandon Cytospin 3 Cytocentrifuge, Fisher Scientific, Erembodegem, Belgium) of BALF cells. After air-drying, the slides were fixed and stained using a fast routine blood cell staining method (Diff-Quick; DQ-ST, MICROPTIC, Barcelona, Spain), and mounted in Entellan (Merck; Overijse, Belgium). Cell types in the BALF cytospins were morphologically characterized under a light microscope and compared between LPS-challenged, sham-treated and untreated controls. Data were used only for general interpretation of the inflammatory effect of instillation, and no further quantification was performed.

### Preparation of live lung slices

Live lung slices were prepared as previously published [60]. In short, WT-Bl6 mice (*n* = 3) were euthanized and the lung tissue was stabilized by slowly instilling a 2% agarose solution (37 °C, low-melt agarose, A4018, Sigma) via a tracheal cannula. After inflation, lungs were dissected and transferred to an ice-cold physiological solution. Precision-cut lung slices (120 μm thick) were sectioned using a vibratome (Microm HM650 V; Microm International, Walldorf, Germany) and 6–8 slices per animal were subsequently kept in the cold physiological solution until further manipulation within the next few hours (maximally 6 h).

### Live cell imaging

As previously reported in detail [8], different cell types in the NEB ME and in the surrounding airway epithelium, such as PNECs, CLCs, CCs and ciliated cells, can be identified in live lung vibratome slices after staining with the fluorescent dye 4-Di-2-ASP (Molecular Probes D-289, Fisher Scientific) and loading with the Ca^2+^ indicator Fluo-4 AM (Molecular Probes F-14202; Fisher Scientific). In short, lung slices were consecutively incubated for 4 min in 4 μM 4-Di-2-ASP in Dulbecco’s modified Eagle’s medium/F-12 (DMEM-F-12; Gibco, Fisher Scientific) at 37 °C, rinsed, and incubated for 1 h at room temperature in 10 μM Fluo-4 AM. For experimental LCI purposes, the lung slices were submerged in a tissue bath (2 ml) mounted on the microscope stage, continuously perfused with physiological solution by a gravity-fed system (flow rate of 0.5 ml/min). Perfusion was paused when BALF was manually pipetted onto the lung slice in the tissue bath. Physiological solution containing a high extracellular potassium concentration ([K^+^]_o_) was prepared by equimolar substitution of KCl for NaCl [8].

High-resolution LCI was performed as extensively described before [8, 13, 60]. In short, a microlens-enhanced dual spinning disk confocal system (Ultra*VIEW* ERS; PerkinElmer, Zaventem, Belgium), equipped with an argon-krypton laser was used. Time-lapse images of changes in Fluo-4 fluorescence (excitation max. 494 nm; emission max. 516 nm) were recorded (2 images/s; 488-nm laser excitation; bandpass 500–560 emission filter) and analyzed off-line by Volocity 2 software (Improvision, Coventry, UK). Regions of interest were manually drawn around identified cell groups of interest, and the fluorescence intensity was plotted against time. Changes in Fluo-4 fluorescence should be interpreted as relative changes in the intracellular Ca^2+^ concentration ([Ca^2+^]_i_). All graphs presented are representative of multiple experiments performed under the respective conditions*.*

### Immunohistochemical staining of lung cryosections for the evaluation of cell division

All mice included in the LPS challenge experiments, including sham-treated and untreated controls, were processed for cryosectioning and immunostaining as detailed below. Mice were euthanized and the lungs were transcardially perfused with physiological solution and subsequently filled with 4% paraformaldehyde (PF) via a tracheal cannula. Lungs, trachea, esophagus and heart were dissected *en bloc*, deaerated in a mild vacuum, and immersion-fixed in the same fixative for 30 min. After rinsing in PBS, tissues were stored overnight in 20% sucrose (in PBS; 4 °C), and mounted in Tissue-Tek O.C.T. (4583; Sakura Finetek Europe, Zoeterwoude, The Netherlands). Consecutive cryostat sections (20 μm thick; Leica CM1950, Diegem, Belgium) of the whole tissue blocks were thaw-mounted on poly-L-lysine-coated microscope slides following a strict pattern (see further on), dried at 37 °C (2 h) and kept at − 80 °C until further use.

Immunohistochemical incubations were performed at room temperature in a closed humidified container. All primary and secondary antisera were diluted in PBS containing 10% normal horse serum and 0.1% BSA (PBS*). Before incubation with the primary antisera, cryostat sections were permeabilized for 1 h with PBS* containing 1% Triton X-100. Sections were then incubated overnight with a combination of the primary antibodies listed in Table 1. For visualization of the immunolabeling, sections were further incubated for 4 h with secondary antibodies (Table 2).

**Table 1:** List of primary antisera used for immunohistochemistry

Primary Antisera	Host	Mc/Pc	Source
Antigen			
Bromo deoxyuridine (BrdU)	Rat	Mc	Abcam ab6326, Cambridge, UK
Calcitonin gene-related peptide (CGRP)	Rabbit	Pc	Sigma C3866, Bornem, Belgium
CGRP	Goat	Pc	Abcam ab36001
Urine protein 1 (UP1)/Clara cell secretory protein (CCSP)	Rabbit	Pc	Abcam ab40873

**Table 2:** List of secondary antisera used for immunohistochemistry

Secondary Antisera	Source	Dilution
Antigen		
Cy™5-conjugated Fab fragments of goat anti-rabbit IgG	Jackson ImmunoResearch 111–117-003, West Grove, PA, USA	1/2000
Cy™3-conjugated donkey anti-rat IgG	Jackson ImmunoResearch 712–165-153	1/1000
FITC-conjugated donkey anti-goat IgG	Jackson ImmunoResearch 705–095-147	1/500
Cy™5-conjugated donkey anti-rabbit IgG	Jackson ImmunoResearch 711–175-152	1/500

After a final wash in PBS, the sections were mounted in Citifluor (19,470; Ted Pella, Redding, CA).

For each animal, a few cryosections were routinely stained with hematoxylin and eosin (H&E), dehydrated in a graded series of ethanol and xylene, and mounted in Entellan. Lung sections were evaluated under a light microscope for the histological evaluation of epithelial damage, interstitial inflammation, edema and leukocyte recruitment, as characteristics of pulmonary inflammation and lung injury [61].

A bright-field/epifluorescence microscope (Zeiss Axiophot, Carl Zeiss, Jena, Germany) was used to image the H&E-stained lung cryosections and DiffQuick stained BALF cytospins, and to quickly screen the immunostaining results. All fluorescence images were obtained using a microlens-enhanced dual spinning disk confocal microscope (Ultra*VIEW* VoX; PerkinElmer) equipped with 488 nm, 561 nm and 640 nm diode lasers for excitation of FITC/GFP, Cy3 and Cy5. Images were acquired and processed using Volocity 6.3.1 software (PerkinElmer).

### Data acquisition, quantification and statistics

Quantification of the BrdU-positive cells was performed by manually counting the fluorescent nuclei in the areas of interest. Lung cryosections (20 μm-thick) were collected and selected in a reproducible manner. Per slide, two sections were mounted in such a way that the distance between both sections is 200 μm. In short, ten consecutive sections were mounted on different slides, after which the following 10 sections were mounted in the same order on these slides. The next 20 consecutive sections were collected on 10 new slides, and so on until the lung tissue was completely cut. Then, a first slide for staining was selected based on the presence of airway branches and presumably NEBs. Starting from this slide, six more were taken every 10 slides, thereby avoiding the possibility that more than one section of the same NEB ME could be found and/or counted in the selected slides when immunostained for BrdU and some reference markers. As such, for every mouse in the different treatment groups (LPS-treated, sham treated and untreated control), between 60 and 100 NEBs were visualized under the microscope, by their GFP fluorescence in GAD67-GFP mice or CGRP immunostaining in WT-Bl6 mice, and the PNECs and BrdU-positive cells in the NEB ME were counted.

For each animal in all of the experimental groups, the mean number of BrdU-positive cells per NEB ME was calculated and the data were statistically compared between the different treatment groups, using a nonparametric Kruskal-Wallis test followed by Dunn’s multiple comparisons test. Data are represented as (mean ± SEM).

Potential differences in the number of BrdU-positive cells between the two mouse strains were statistically evaluated using the unpaired t-test for each treatment group, after checking normal distribution of the counts.

## Results

### Evaluation of the pulmonary effects of low dose LPS challenge

Although the recorded plethysmographic data did not qualify for quantification, due to individual variation inherent to the use of unrestrained young mice, some of the observations were of importance for the presented study. Apart from clear but variable differences in the measurements of TE, RT, EIP and TV between untreated controls and LPS-challenged (and to a lesser extent also sham-treated) mice during the first 2 to 6 h, plethysmography could no longer distinguish LPS-challenged from untreated animals 8 h or longer after treatment (data not shown).

**Fig. 1:**
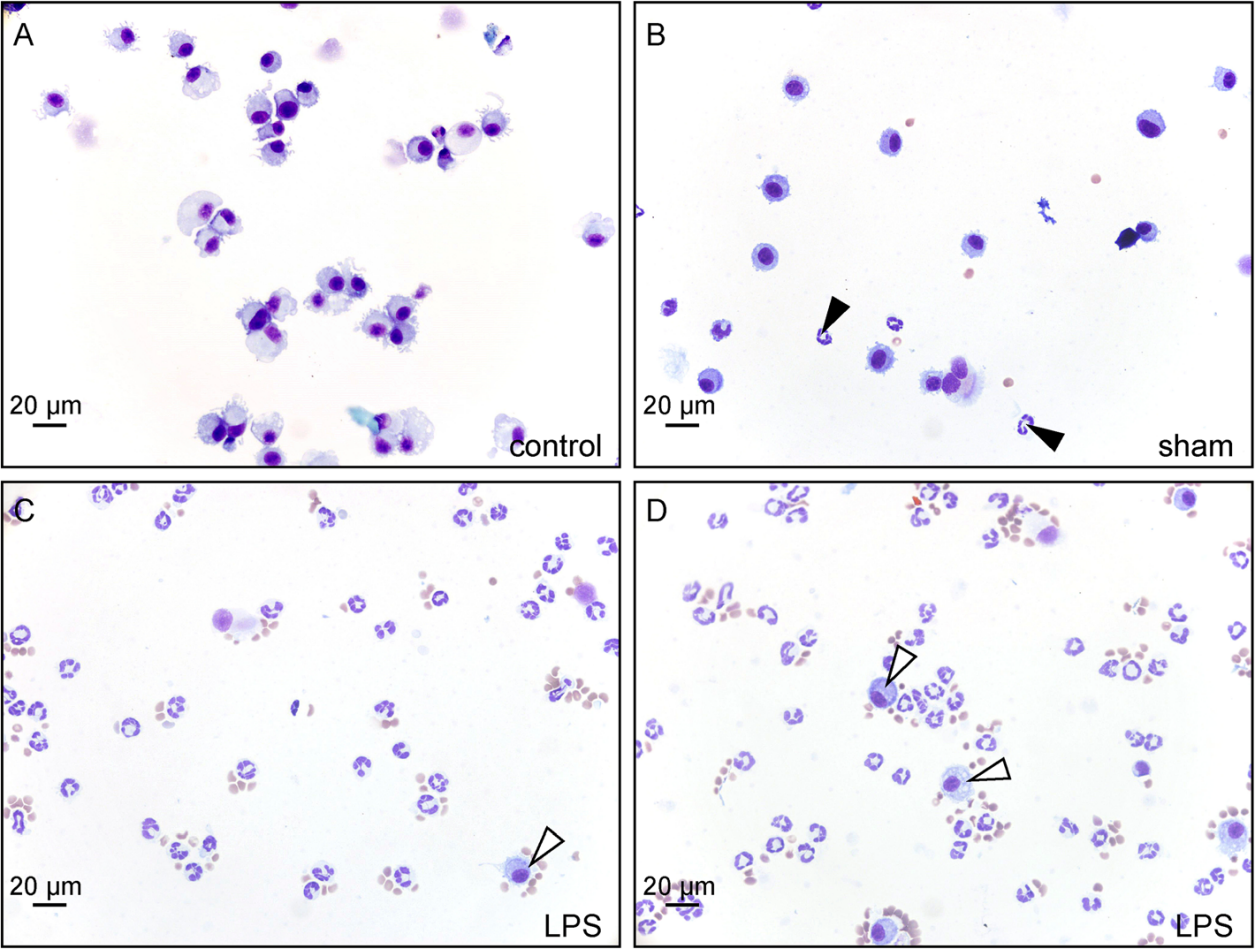
Representative images, with similar cell densities, of Diff-Quick-stained cytospin preparations of BALF collected from mice that received no instillation (untreated healthy control animal; **a**), 16 h after an intratracheal instillation with 0.9% NaCl (sham-treatment; **b**), or with LPS (**c**, **d**). Cell densities of the cytospin preparations were ‘normalized’ between the experimental groups and are therefore unrelated to the initial cell numbers in the BALF. **a** BALF of a healthy control mouse contains virtually no other cells than macrophages (acentric oval nuclei and bluish cytoplasm). **b** Some neutrophils (segmented nuclei and unstained cytoplasm; *arrowheads*) appear to be infiltrated in the airways after a sham instillation but macrophages still constitute the majority of cells in this sample. **c**, **d** Massive neutrophil influx is seen as a response to the LPS instillation, and consequently a relatively low number of macrophages (*open arrowheads*). Note that the preparations of treated animals also harbor some red blood cells (small brownish dots)

To assess possible inflammatory changes in the airway environment, BALF was collected from the same animals that had been monitored by plethysmography (16 h after instillation of LPS or saline and untreated), and processed for the generation of cytospin preparations. While BALF of healthy control animals showed macrophage-like cells only (Fig. 1a), the majority of leukocytes in the LPS-treated mice appeared to be neutrophils (Fig. 1c, d). Neutrophils were also seen in BALF of the sham-treated mice (Fig. 1b), but clearly to a lesser extent than after LPS challenge.

**Fig. 2:**
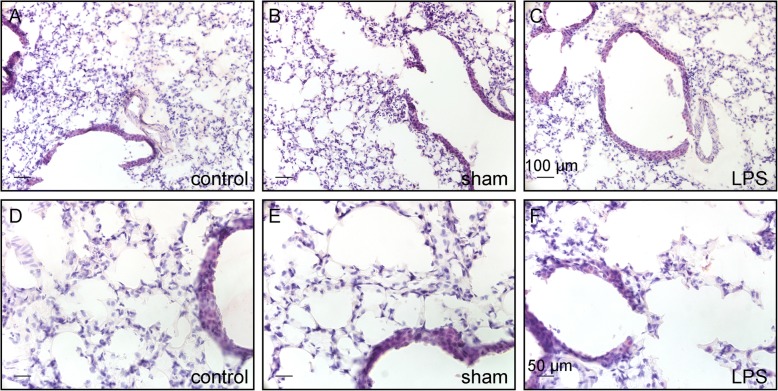
Representative images of HE-stained lung cryosections from untreated control (**a**, **d**), and 48 h after intratracheal instillation with 0.9% NaCl (sham-treatment; **b**, **e**), or with LPS (**c**, **f**). Note that lung morphology shows no clear histological differences between the groups

The applied single low-dose intratracheal LPS challenge did not cause obvious histological changes in the airway epithelium or alveolar areas in H&E-stained lung sections (Fig. 2).

An important element for the interpretation of the following experimental data is that this combined approach (plethysmography, evaluation of the collected BALF; lung histology), supports the idea that the applied single low dose of intratracheal LPS induces a mild and transient inflammation.

### Application of BALF from LPS-challenged mice to the NEB ME of healthy control mice

**Fig. 3:**
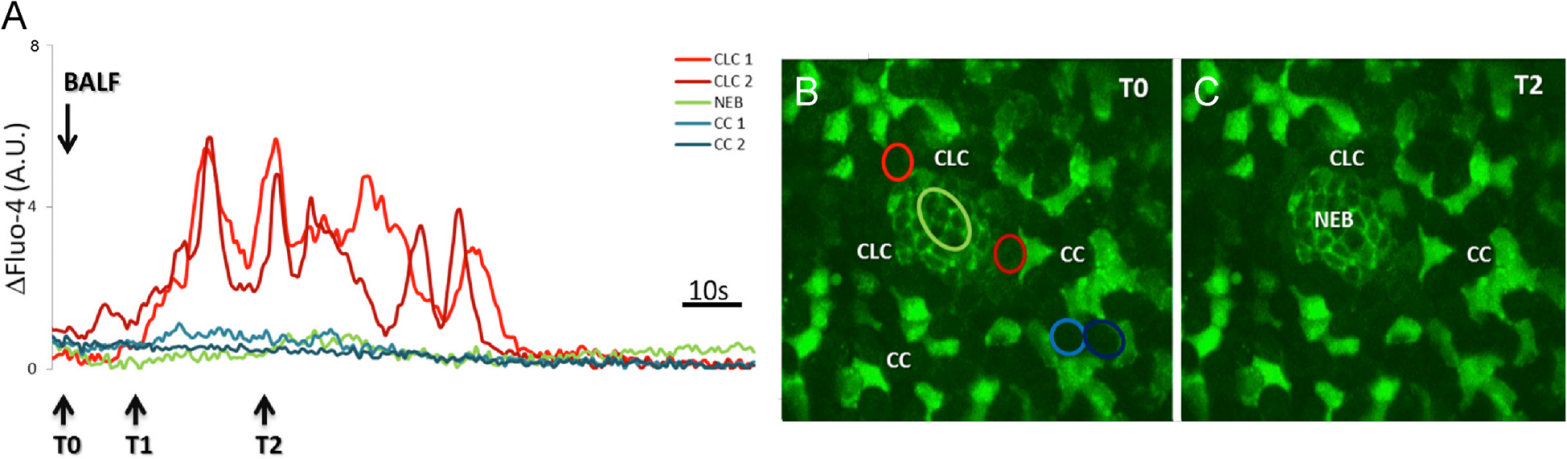
LCI representation of the effects of application of BALF of an LPS-challenged mouse to the NEB ME in a vibratome cut live lung slice of a control mouse. **a** Graphs plotting the time course of Fluo-4 fluorescence intensity, as an indicator for [Ca^2+^]_i_, in different cell types in the airway epithelium. To facilitate interpretation, grey levels of each region of interest (ROI) were adjusted for the basal level of (background) fluorescence at the start of imaging. After application of BALF (=T0), CLCs that surround the NEB cells show a calcium-mediated activation within about 10 s (=T1). The oscillating [Ca^2+^]_i_ rise in CLCs continues (=T2) for more than a minute post-exposure. No changes in [Ca^2+^]_i_ can be observed in NEB cells or CCs. **b**, **c** Corresponding pseudo-color time-lapse images of Fluo-4 fluorescence at two time points (B = T0; C = T2) after the application of BALF. Note that the ROIs corresponding to the graphs in (**a**) are represented in the same color code in image **b**

Apart from detecting/recording [Ca^2+^]_i_ fluctuations in the NEB ME, freshly cut live lung slices co-loaded with 4-Di-2-Asp and Fluo-4, also allowed to differentiate between all cell types in the NEB ME and the surrounding airway epithelium (for detailed explanation see [8]). In short, very small and moderately fluorescent grouped NEB cells are encircled by a virtually non-fluorescent rim that harbors much larger CLCs, and are further surrounded by intermingled strongly fluorescent polygonal ciliated cells and large rounded unstained CCs (Fig. 3b).

To allow fast evaluation of the effect of LPS-induced inflammatory mediators on the NEB ME, BALF that had been collected from mice 16 h after intratracheal instillation of LPS, was applied to lung slices of healthy control mice in the course of the LCI experiments.

BALF of LPS-treated mice was seen to induce a reversible and reproducible [Ca^2+^]_i_ rise, selectively in CLCs of the NEB ME (Fig. 3). CLCs appeared to react within about 10 s after administration of BALF and revealed [Ca^2+^]_i_ oscillations for about a minute. On the other hand, NEB cells, CCs and ciliated cells did not show a [Ca^2+^]_i_ rise. Comparable challenge of lung slices with LPS (15 μg/ml in physiological solution) or with BALF of sham and untreated control mice did not induce a [Ca^2+^]_i_ rise in any cell type in the NEB ME or airway epithelium, while the physiological responsiveness of the lung slices could be confirmed by the typical fast and reversible [Ca^2+^]_i_ increase in NEB cells and a slightly delayed [Ca^2+^]_i_ rise in CLCs, following a 5-s application of 50 mM K^+^ (not shown) [8].

### Effects of a single intratracheal instillation with LPS and the resulting transient mild inflammation on the NEB ME

The above observation that CLCs –– which are presumed quiescent airway epithelial stem cells in healthy control mice –– show a calcium-mediated activation upon short-term application of BALF that contains soluble mediators from the airways of LPS-challenged mice, raised the question as to what the effects might be on CLCs that experience long-term exposure to these mediators in airways of LPS-treated mice. To follow up on the hypothesis that activation of quiescent (non-dividing) stem cells might be linked to proliferation, the potential effect of intratracheal administration of a single low dose of LPS on airway epithelial cell proliferation in general, and on the NEB ME in particular, was monitored using simultaneous BrdU incorporation as a marker for cells that divide during the experimental window. Three experimental groups were included: untreated control mice, mice receiving an intratracheal instillation with LPS, and mice with an intratracheal instillation of a 0.9% NaCl solution (= sham control).

**Fig. 4:**
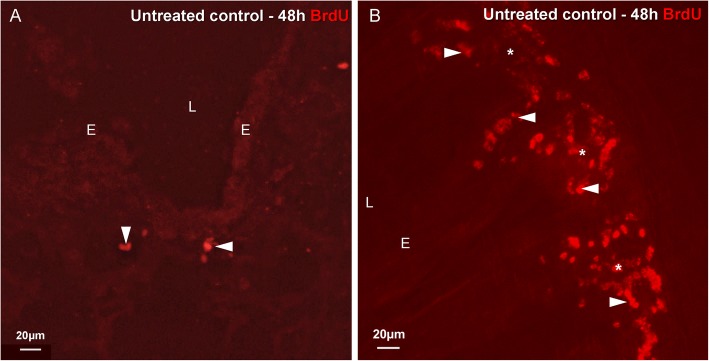
Immunostaining for BrdU (*red* Cy3 fluorescence) in cryosections of an intrapulmonary airway (**a**) and small intestine (**b**) of the same mouse that received no treatment, except for the BrdU injections (i.p.), 48 h and 24 h prior to sacrifice. **a** The airway epithelium rarely harbors divided BrdU-positive cells. The majority of BrdU-labeled nuclei (*arrowheads*) are located in subepithelial layers. **b** In the crypts (*asterisks*) and the basal parts of the villus epithelium of the small intestine, a large number of BrdU-labeled nuclei (*arrowheads*) –– i.e., originating from cells that have divided during the 48 h experimental window –– can be observed. *L: airway lumen, E: airway epithelium*

In cryosections of the lungs of untreated healthy 3-week-old WT-Bl6 mice, hardly any divided cells were discerned in control airway epithelium (CAE) within the investigated time windows (24, 48 (Fig. 4a) or 72 h) as confirmed by BrdU labeling. The very rare divided epithelial cells appeared to be randomly distributed. The majority of cells with BrdU-labeled nuclei in the airways of these untreated controls were found subepithelially (Fig. 4a). As a positive control, BrdU incorporation was evaluated in sections of a piece of small intestine that was additionally collected from each mouse. A large number of divided (BrdU-labeled) epithelial cells was invariably observed in crypts of the intestinal villi (Fig. 4b).

**Fig. 5:**
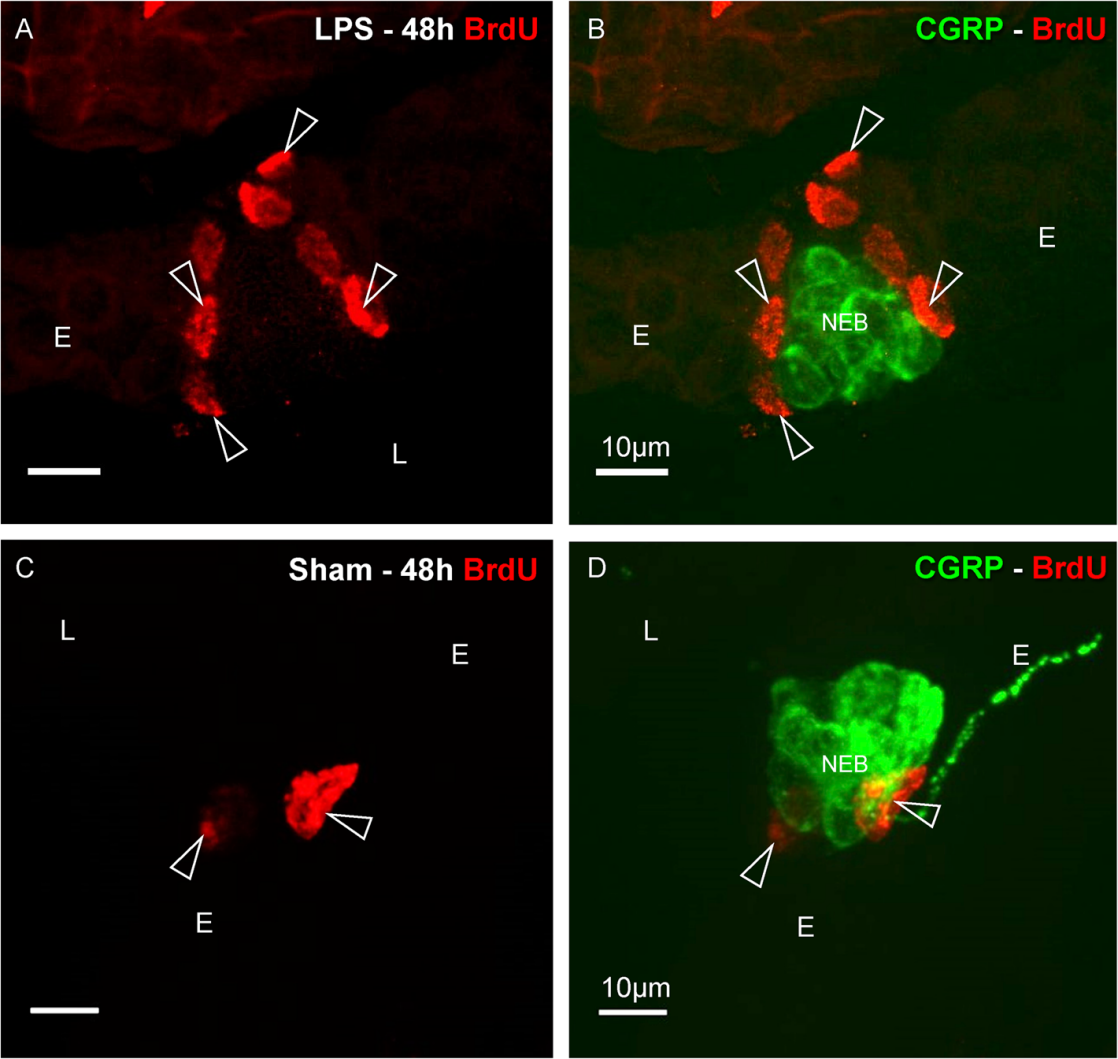
Immunostaining for BrdU (*red* Cy3 fluorescence) and CGRP (*green* FITC fluorescence) in intrapulmonary airways 48 h after an intratracheal LPS (**a**, **b**) or sham instillation (**c**, **d**) in WT-Bl6 mice. **a** After LPS challenge, clustered BrdU-positive (divided) cells are observed in the epithelial layer (*open arrowheads*). **b** Additional CGRP immunostaining reveals that the intraepithelial BrdU-labeled cells are typically grouped around NEBs. **c**, **d** After a sham instillation, intraepithelial BrdU-positive cells also appear to be located in the neighborhood of NEBs (*open arrowhead*), but are less numerous than after LPS treatment. *L: airway lumen, E: airway epithelium*

Forty-eight hours after a single intratracheal instillation with low-dose LPS, a considerable number of divided (BrdU-positive) epithelial cells was detected, typically clustered in distinct areas of the airway epithelium of WT-Bl6 mice (Fig. 5a).

In sham-treated WT-Bl6 mice, BrdU-positive cells also appeared to be more numerous at specific locations in the airway epithelium (Fig. 5c) than in untreated controls, although the increase was less pronounced than that seen in LPS-treated mice.

Double immunostaining of the BrdU-labeled sections for CGRP, as a marker for NEBs, revealed that the great majority of divided airway epithelial cells were located in the NEB ME following LPS challenge (Fig. 5b, d).

All of the above data were obtained using 3-week-old mice, but a similar selective cell proliferation (BrdU positive nuclei) in the NEB ME was observed in LPS-challenged adult mice (not shown).

**Fig. 6:**
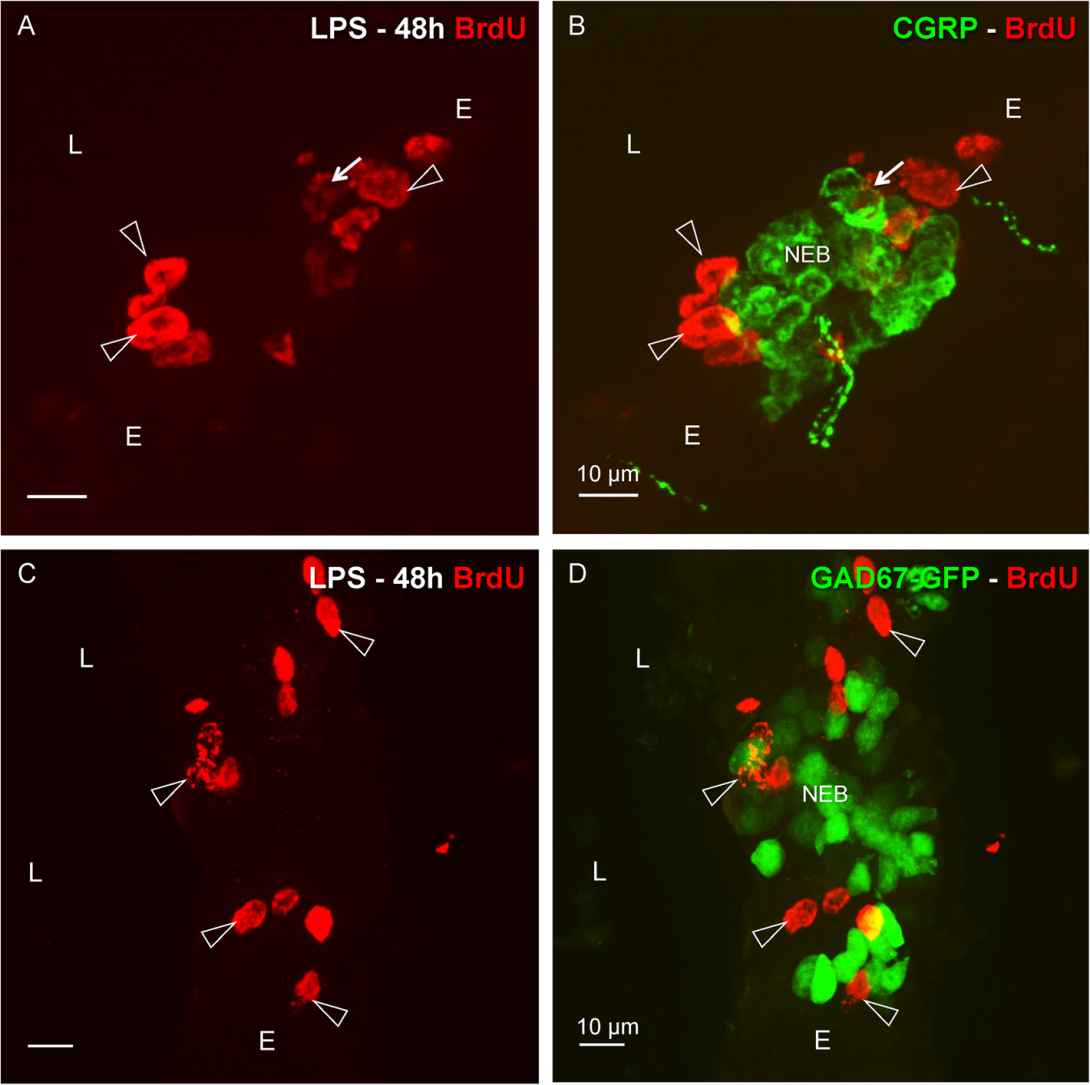
Comparison of the distribution of BrdU-labeled (*red* Cy3 fluorescence) airway epithelial cells 48 h after LPS challenge of WT-Bl6 (**a**, **b**) and GAD67-GFP mice (**c**, **d**). **a**, **c** Clustered BrdU-positive nuclei (*open arrowheads*) can be observed at distinct locations in the airway epithelium. Combination with CGRP immunostaining (**b**; *green* FITC fluorescence) for WT-Bl6 mice, or visualization of GFP-fluorescent NEB cells (**d**) in GAD67-GFP mice, reveals that the majority of divided cells that have incorporated BrdU are found in the immediate neighborhood of NEBs. No obvious differences can be seen between WT-Bl6 and GAD67-GFP mice. Note that only very occasionally, a BrdU-positive nucleus can be seen in a NEB cell (*arrow;* **a***,* **b**)*. L: airway lumen, E: airway epithelium*

Given the apparent close link between NEBs and divided (BrdU-labeled) epithelial cells in the airways of LPS-challenged mice, the use of GAD67-GFP mice that harbor intrinsically GFP-fluorescent NEBs [58] would offer considerable advantages for further quantification. Therefore, we verified whether similar results could be obtained in WT-Bl6 and GAD67-GFP mice. Comparable to what was seen using WT-Bl6 mice with CGRP as a NEB marker (Figs. 5a, b and 6a, b), LPS-treated GAD67-GFP mice also showed BrdU-labeled (divided) airway epithelial cells that were selectively grouped around GFP-fluorescent NEBs (Fig. 6c, d).

Both in healthy controls, sham-treated and LPS-challenged mice, the numbers of CGRP- or GFP-positive PNECs with BrdU-positive nuclei were very low after 48 h exposure.

Since the majority of proliferating (BrdU-labeled) airway epithelial cells in LPS-treated mice was located in the NEB ME, closely surrounding NEB cells, and because BALF of LPS-challenged mice was seen to selectively activate CLCs in the NEB ME, we further investigated whether BrdU-positive cells co-label with markers for CCs/CLCs.

### Characterization of the cell type(s) that typically divide in the NEB ME following LPS challenge

**Fig. 7:**
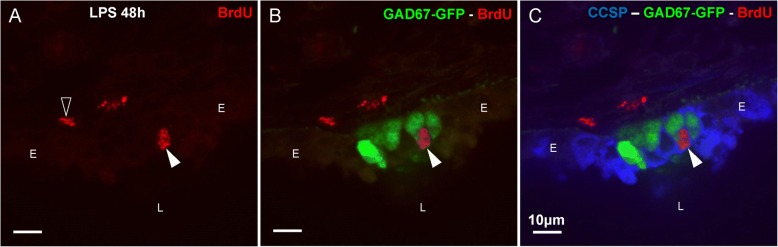
Single confocal optical section of the airway epithelium in a cryosection of the lungs of a GAD67-GFP mouse 48 h after challenge with LPS. **a** BrdU-positive nucleus (*arrowhead; red* Cy3 fluorescence) in the airway epithelium, and some subepithelial BrdU staining (*open arrowhead*). **b** Combination with GAD67-GFP fluorescence (*green*), marking PNECs in the NEB ME. **c** Image of the three channels. In the same section, CCs/CLCs are immunostained for the Clara cell-specific protein CCSP (*blue* pseudo-color of Cy5 fluorescence), and are seen to surround GFP-labeled NEB cells. Note that the divided epithelial cell (BrdU-labeled nucleus; *arrowhead*) is located adjacent to the PNECs and co-stained with CCSP, and can therefore be identified as a CLC. *L: airway lumen, E: airway epithelium*

To further confirm the identity of BrdU-labeled cells in the NEB ME as CLCs, lung sections (48 h after LPS instillation) were additionally immunostained for CCSP, a marker of both CCs and CLCs surrounding NEB cells. CCSP was used due to the lack of a selective marker for CLCs in postnatal lungs and revealed that cells with BrdU-labeled nuclei in the NEB ME invariably co-stained for CCSP (Fig. 7c), confirming that mainly CLCs were concerned.

### Selection of the most relevant time window to study cell proliferation in the NEB ME following LPS challenge

Because single intratracheal instillation of LPS appeared to specifically induce cell division (BrdU incorporation) in the NEB ME in a 48 h time window, we next evaluated whether this was the most relevant time point for visualization/quantification of cell proliferation in the NEB ME. To this end, three time windows were included in a pilot experiment; lungs were collected 24 h, 48 h and 72 h after LPS challenge. Our preliminary data revealed BrdU-labeled airway epithelial cells in the NEB ME already after 24 h, although the effect appeared to be much more prominent 48 h after LPS challenge (Figs. 5a,b, 6 and 7). After 72 h, some of the BrdU-labeled epithelial cells were located a few cells away from NEBs, making this time window somewhat less selective for the NEB ME.

**Fig. 8:**
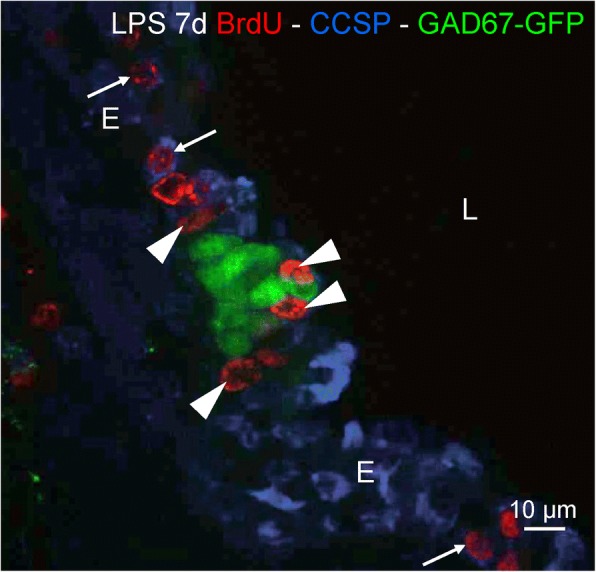
Distribution of divided (BrdU-stained; *red* Cy3 fluorescence) airway epithelial cells seven days after LPS challenge. Similar to the 48 h time window, several CCSP-immunostained (*blue* pseudo-color of Cy5 fluorescence), BrdU-positive nuclei (*arrowheads*) can be observed specifically surrounding GAD67-GFP fluorescent (*green*) PNECs in the NEB ME. Note, however, the additional presence of CCSP-stained BrdU-labeled CCs in the surrounding airway epithelium (*open arrowheads*). *L: airway lumen, E: airway epithelium*

Interestingly, 7 days after LPS challenge, BrdU-positive nuclei could still be detected in CCSP-positive CLCs in the NEB ME, similar to what was seen after 48 h, but also in CCSP-labeled CCs in the surrounding airway epithelium (Fig. 8).

Because the NEB ME is the main area of interest for the present investigation, and considering the observed calcium-mediated activation of CLCs, the time window of 48 h BrdU incorporation after LPS challenge was chosen for further quantification of cell proliferation in the NEB ME.

### Quantitative analysis of BrdU-labeled cells in the NEB ME after LPS treatment

#### Evaluation of potential differences between WT-Bl6 and GAD67-GFP mice

Cell division was quantified in a 48 h time window using WT-Bl6 and GAD67-GFP mice that had been injected with BrdU at time point zero and after 24 h. Three groups were included, i.e., mice receiving a single intratracheal injection with either LPS or 0.9% NaCl at time point zero, and untreated control mice.

To avoid that unlikely variations in cell proliferation between WT-Bl6 mice and GAD67-GFP mice would hamper the interpretation of observed differences, both mouse strains were included in the quantifications.

The significance of possible variations between GAD67-GFP (*n* = 5) and WT-Bl6 (n = 5) mice was evaluated by means of a nonparametric t-test on the percentages of NEBs that showed BrdU-positive cells for the two treatment groups (LPS-challenged WT-Bl6: 72.2 ± 4.4% vs. LPS-challenged GAD67-GFP mice: 61.9 ± 2.1%, *p* = 0.1; sham-treated WT-Bl6: 23.2 ± 2.6% vs. sham-treated GAD67-GFP mice: 19.3 ± 3.5%, *p* = 0.4) and on the mean number of BrdU-positive cells per activated NEB ME (LPS-challenged WT-Bl6: 3.45 ± 0.37 vs. LPS-challenged GAD67-GFP mice: 3.30 ± 0.26, *p* = 0.1; sham-treated WT-Bl6: 1.54 ± 0.53 vs. sham-treated GAD67-GFP mice: 1.78 ± 0.07, *p* = 0.4). Consequently, data for the two strains could be pooled for further quantification of the potential differences between the experimental groups.

#### Correlation between the number of BrdU-labeled cells and the number of PNECs in NEBs

**Fig. 9:**
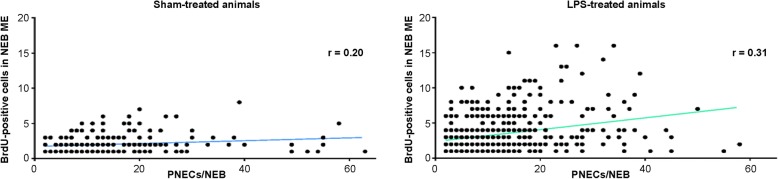
Graph plotting the number of BrdU-positive (divided) cells per NEB ME as a function of the number of PNECs for each NEB that harbors BrdU-positive cells, both in sham- (left graph) and LPS-treated (right graph) mice (*n* = 10; 5 WT-Bl6 and 5 GAD67-GFP mice for each treatment group). The full data sets show no significant positive correlation between the size of NEBs and the number of BrdU-positive cells in their ME. *r = Spearman correlation coefficient*

To examine whether the size (number of PNECs) of NEBs is important for the number of divided cells in the NEB ME, the number of BrdU-positive cells was plotted out against the number of PNECs for each counted NEB of all animals in the treatment groups. Although the mean number of PNECs per NEB (10.8 ± 0.6 for LPS-treated animals vs. 11.3 ± 1.0 for sham-treated) was, as expected, not significantly different for both groups (unpaired t-test; *p* > 0.66), the mean number of BrdU-positive cells per NEB ME (0.5 ± 0.1 for sham-treated vs. 2.1 ± 0.3 for LPS-treated mice) was significantly higher (*p* < 0.0005) in the LPS-challenged mice (Fig. 9).

Overall, larger NEBs (more PNECs) appeared to harbor in their ME a modestly higher number of cells that have divided during a 48 h time window, although only a small non-significant positive trend in the correlation between the number of PNECs and the number of divided cells in the NEB ME was observed for the complete set of quantified NEBs in both treatment groups (sham-treated: *r* = 0.20; LPS-treated mice: *r* = 0.31; Fig. 9).

Further quantifications were therefore carried out without additional analysis of the number of PNECs in each NEB.

#### Percentage of NEBs that harbor divided cells in their ME

**Fig. 10:**
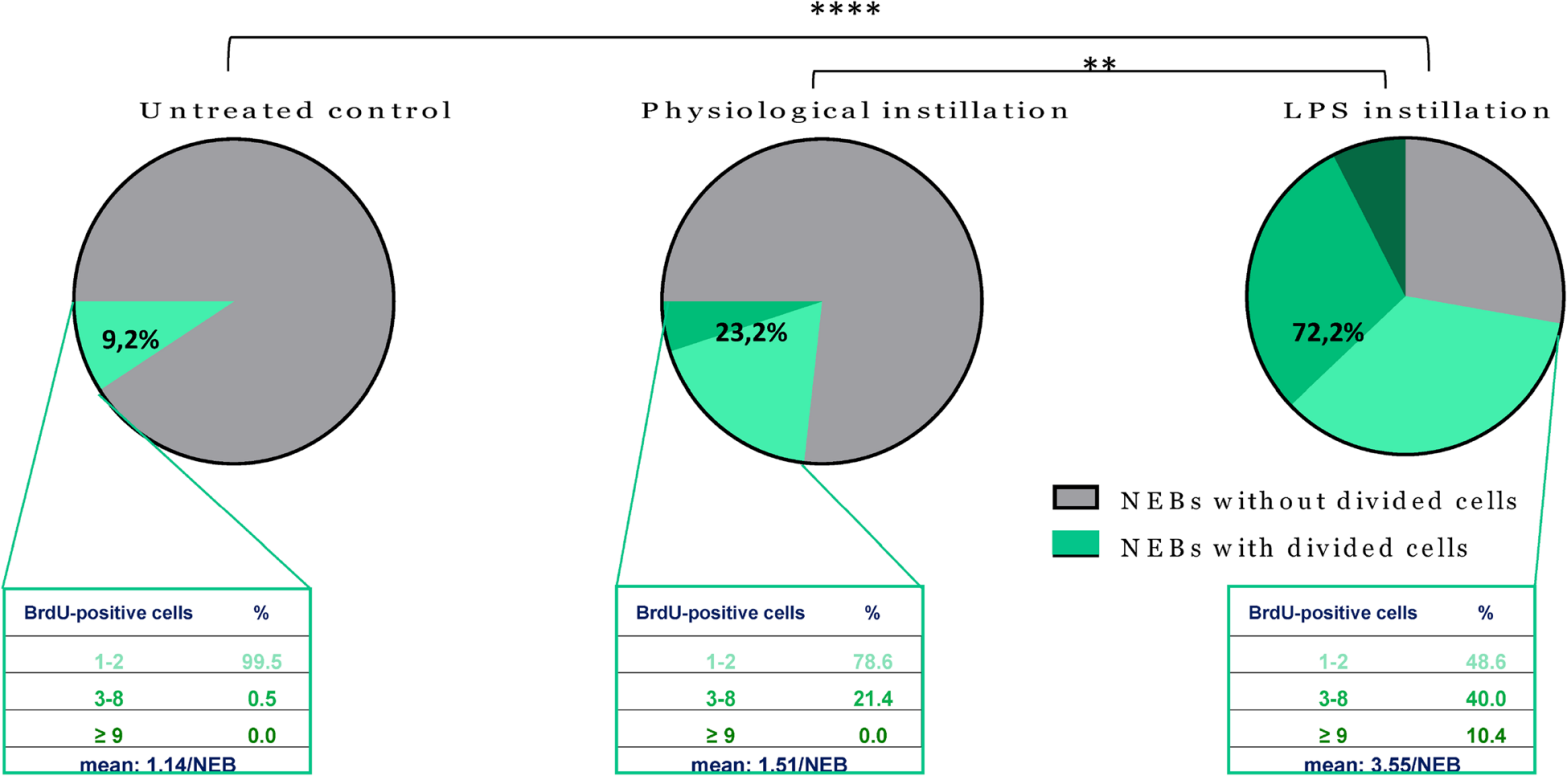
Percentage of NEBs with cells in their ME that have divided during the 48 h experimental window. In the untreated controls (*n* = 5), less than 10% of the NEB MEs harbor divided cells. Interestingly, both the percentage of NEBs with BrdU-positive cells (about 23%) and the number of divided cells per NEB ME is higher in sham-treated mice (n = 10) than in untreated control mice. After LPS instillation (n = 10), however, about 72% of the NEBs show divided cells in their ME. The green part of the pie charts represents the percentage of NEBs with BrdU-positive cells, and is further subdivided based on the number of BrdU-labeled cells per NEB. The data in the framed areas represent the percentages of NEBs with one or two, three to eight, or nine and more BrdU-positive cells. The mean number of BrdU-positive cells is significantly different (Dunn’s multiple comparisons test) between the untreated control and the LPS-treated group (****, *p* < 0.0001) and between the sham- and LPS-treated group (**, *p* < 0.01)

Extensive quantification of BrdU-labeled (divided) cells in the NEB ME in the airways of LPS-challenged, sham-treated and untreated controls revealed clear differences between the experimental groups (Fig. 10). Mice that received an intratracheal LPS instillation harbored a significantly higher mean percentage (Non-parametric Kruskal-Wallis test) of NEBs with BrdU-positive cells in their ME (72.2 ± 2.4%), as compared to sham-treated animals (23.2 ± 2.6%) and untreated control mice (9.2 ± 2.4%).

#### Number of divided cells per NEB ME

A closer look at the number of BrdU-stained cells (framed areas in Fig. 10; Table 3) did not show clustering of divided cells in the NEB ME in untreated control mice (mean number of BrdU-positive cells for all NEBs with divided cells: 1.14 ± 0.3 BrdU-positive cells/NEB ME; total of 10.5 BrdU-positive cells/100 NEBs). In the sham experiments, relatively more NEBs showed divided cells in their ME, but similar to the untreated controls they were mainly observed as solitary BrdU-stained cells (mean number: 1.51 ± 0.26 BrdU-positive cells/NEB ME; total of 35 BrdU-positive cells/100 NEBs). However, clustering of divided cells in the NEB ME was seen after LPS treatment, with 40% of the NEBs harboring three to eight BrdU-stained cells and 10% of the NEBs even nine or more (mean number: 3.55 ± 0.6 BrdU-positive cells/NEB ME; total of 256.3 BrdU-positive cells/100 NEBs).

**Table 3:** Representation of the total number of BrdU-positive cells in the NEB ME

Untreated control											
Total number of BrdU^+^ cells		11		8		11		9		0	
NEB ME with ≤2 BrdU^+^ cells		6		6		6		7		0	
≥2 BrdU^+^ cells	≥9 BrdU^+^ cells	1	0	1	0	0	0	0	0	0	0
Sham-treated											
Total number of BrdU^+^ cells		16		61		54		21		15	
NEB ME with ≤2 BrdU^+^ cells		10		21		25		10		9	
≥2 BrdU^+^ cells	≥9 BrdU^+^ cells	0	0	9	0	4	0	1	0	0	0
LPS-treated											
Total number of BrdU^+^ cells		187		316		135		130		190	
NEB ME with ≤2 BrdU^+^ cells		13		33		32		30		49	
≥2 BrdU^+^ cells	≥9 BrdU^+^ cells	4	20	36	6	21	0	9	2	20	1

The table includes both the total numbers of BrdU-positive cells in the NEB ME per mouse (*n* = 5; GAD67-GFP mice) for each experimental group, and a separation of NEB MEs in different subgroups, i.e., NEB ME with ‘mainly’ single (two or less) BrdU-labeled cells and NEB ME with higher numbers of divided cells (three to eight, or nine and more)

Overall (Fig. 10 and Table 3), quantitative analysis revealed that the NEB MEs in untreated controls typically display a very low number of BrdU-positive airway epithelial cells, indicating that virtually no cells have divided during the 48 h experimental window. The manipulation of intratracheal instillation (0.9% NaCl; sham-control) on its own appeared to induce a limited but enhanced cell division in the NEB ME, whereas the percentage of NEBs with BrdU-stained cells in the NEB ME and the total number of divided cells were remarkably higher after LPS instillation.

### Subdivision of BrdU-labeled cell types in the NEB ME

**Fig. 11:**
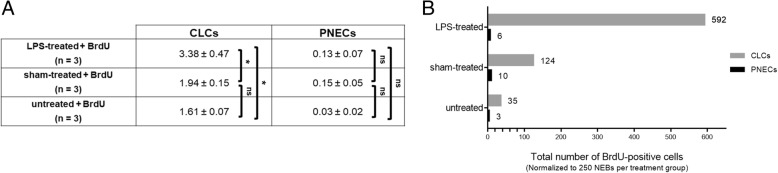
Table providing the mean numbers of divided (BrdU-labeled) CLCs and PNECs per activated NEB ME (**a**) and graph representing the total numbers of BrdU-positive CLCs and PNECs (**b**) for all quantified NEBs in the three experimental groups (*n* = 3 GAD67-GFP mice per group)

For a limited number of experiments (*n* = 3 GAD67-GFP mice per group), all BrdU-labeled cells of each NEB ME were individually identified –– as CLCs or PNECs –– and counted (data shown in Fig. 11). Clearly, the number of divided PNECs was very limited compared to that of CLCs in the NEB ME. Moreover, the mean number of BrdU-labeled PNECs was not significantly different between the three experimental groups (Kruskal-Wallis; *p* = 0.25).

These additional data further confirmed the significantly higher number of divided CLCs in the LPS-treated group (Kruskal-Wallis; *p* = 0.01).

## Discussion

Despite having a very low cellular turnover rate in healthy conditions, the lung and airway epithelium are capable of responding quickly to acute injury, due to the presence of a restricted set of quiescent stem cells that are able to re-enter the cell cycle [4]. Detailed knowledge of the molecular characteristics, silencing and activation pathways of these specific types of stem cells will be essential for a good understanding of the possibilities for lung regeneration after injury. It is, however, very challenging to study rare cell populations with potential but ‘dormant’ stem cell characteristics in healthy lungs. Subject of the present study are poorly studied potential airway stem cells that are selectively located in the pulmonary NEB ME. We developed a highly reproducible and minimally invasive model for transient lung inflammation, based on a single low-dose intratracheal LPS instillation.

The bacterial endotoxin LPS is commonly used to induce a pulmonary inflammatory response because it is easy to administer and it tends to result in a reproducible lung injury (for review see [57]). According to literature data, intratracheal instillation with doses of 0.2 mg LPS/kg BW or less may be regarded as physiological [52], whereas 5 mg LPS/kg BW results in a moderate reversible lung injury [62, 63], which prompted us to apply 1 mg LPS/kg BW in the presented model.

Unrestrained whole-body plethysmography revealed that respiratory functions –selected parameters were Te, RT, EIP and TV– were compromised during the first two to six hours following a single low-dose LPS challenge, but also that these physiological parameters returned to the level of untreated control mice within 8 h after treatment.

Cytospin preparations of BALF, collected 16 h after LPS treatment, showed many neutrophils, indicative of an inflammatory reaction. This observation is in line with literature data showing that intratracheal LPS exposure resulted in a pulmonary infiltration of neutrophils as early as 4 h after challenge [51]. At the time point of evaluation of cell division, i.e., 48 h after LPS instillation, the morphology of the airway epithelium could not be distinguished from that in untreated control mice, as shown in H&E-stained lung cryostat sections.

It can be concluded that the intratracheal LPS challenge used in the present study does initiate an early influx of neutrophils, reminiscent of airway inflammation, but that the resulting injury is very mild and transient. The latter aspect is in contrast to the severe lung injury models that so far have been used in the majority of studies dealing with the activation of dedicated airway epithelial stem cell niches [44, 57], and will be discussed further on.

Cell-free BALF has been reported to harbor elevated cyto- and chemokine levels in the early phase (4 h–24 h hours) after intratracheal LPS instillation [52]. In the later phase (24-48 h post-instillation) these cytokine concentrations normalize, although the number of neutrophils remains elevated for at least 72 h [52].

To evaluate the potential effects of soluble mediators in BALF on healthy mouse airway epithelium, we collected BALF of LPS-challenged mice 16 h post-instillation. Acute short-term application of this BALF to the airway epithelium, and in particular to the NEB ME, in ex vivo lung slices of control mice in the course of LCI experiments, revealed a reversible and reproducible selective calcium-mediated activation of CLCs, but not of NEB cells, CCs or ciliated cells. Since administration of BALF of control mice (sham-treated and untreated), or of an LPS-containing solution, in our settings failed to mimic the activation of CLCs, it is reasonable to assume that one of the soluble inflammatory mediators released by activated macrophages and/or infiltrated neutrophils in the BALF may trigger the observed calcium-mediated activation of CLCs. So far, the identity of the molecule that is responsible for activation of the CLCs remains unknown and needs further investigation, which was not the focus of the present study.

Logically assuming that the NEB MEs in LPS-challenged mice are continuously exposed to bronchoalveolar lining fluid that harbors the same mediators as BALF, the presented study was designed to find out what could be the effect on CLCs as a potential quiescent stem cell population.

Mainly during fetal and early postnatal life, stem cell characteristics have been ascribed to CLCs [12, 30, 64–67]. Lineage-tracing models suggest that CLCs have the capacity to self-renew [12, 42]. Clara cell-like precursors appear to generate both Clara and ciliated cells during development and repair, driven by Notch signaling [45, 46], and have been suggested to contribute to homeostasis of the intrapulmonary airway epithelium [35, 42].

Full depletion of progenitor CCs and lineage tracing have resulted in the identification of a rare label-retaining and self-renewing subpopulation of CCs in the NEB ME [68], referred to as CLCs in the present study, which behave as airway epithelial stem cells and/or are critical for stem cell maintenance [12]. Both regeneration and tumor formation may be directed by CCs or at least subpopulations of CCs with progenitor or stem cell characteristics [50, 69–71].

Until now, it has been believed that severe injury is required to activate dedicated airway epithelial stem cell niches –such as the NEB ME– for repair, while replacement of epithelial cells after mild injury (including homeostasis) is thought to be managed by the large pool of general CCs [2]. Interestingly, we show here that mild injury under the right conditions definitely can result in the selective activation of silent CLCs in the NEB ME and not of general CCs.

Our observation (both from LCI and cell proliferation studies) of the activation of dedicated airway epithelial stem cells, in the apparent absence of damage, suggests that early mediators of the induced inflammation may be directly involved in activating the stem cells. This idea is supported by recent publications pointing out the emerging role of neutrophils in repair after lung injury, highlighting the essential contribution of neutrophils –which can transmigrate into the airspaces within minutes after the initial insult– in both early injury and repair (for review see [72]).

Incorporation of the thymidine analogue BrdU in DNA during the S-phase of cell division is a commonly used method to follow cell proliferation in a selected experimental time window [73, 74]. In the present study, BrdU incorporation for 48 h and subsequent BrdU immunostaining in cryostat sections, revealed that cell division was very rare in airway epithelium of untreated control mice, while a large number of BrdU-positive (divided) cells were present in the epithelium of small intestinal villi. These observations are in accordance with literature reports for rodents, with an estimated life time of about 4 days for enterocytes in the small intestine, whereas the turnover time of postnatal airway epithelium is more than 100 days [47]. Although some differences in proliferation of airway epithelial cells –probably due to health and pathogen status, strain and age of individual animals– have been reported (for review see [75]), cell division remains remarkably low in the absence of injury [4].

In our experimental setting, combined LPS challenge and BrdU incorporation resulted in a strongly enhanced proliferation of epithelial cells, the majority of which are typically grouped around NEB cells 48 h after LPS challenge. At that time point, the number of divided cells in the NEB ME was found to be 24.6 fold higher in LPS-treated mice than in untreated controls (256.3 vs. 10.4 BrdU-positive cells/100 NEBs), with some NEBs showing up to 24 divided cells in their ME niche. Quantification of the divided cells for each NEB ME and comparison between the LPS-treated and untreated control group showed that LPS challenge induced a highly significant proliferation of cells surrounding the NEBs, i.e., more than 72% of all counted NEBs harbored divided cells in their ME after 48 h as opposed to less than 10% in control mice. Double staining for CCSP, a marker for CCs and CLCs, as well as the location of the proliferated cell type were used to identify the majority of divided cells in the NEB ME as CLCs. The observation that CCSP-positive, BrdU-retaining CLCs were still found in the NEB ME 7 days after LPS challenge, in addition to the presence of CCSP-positive BrdU-labeled CCs dispersed in the airway epithelium surrounding NEBs, strongly implicates CLCs in the NEB ME as a population of self-renewing stem cells.

Although the adopted single low-dose intratracheal LPS challenge mouse model was shown to cause only mild and transient inflammation, it did induce a prominent selective proliferation of CLCs in the NEB ME.

No significant correlation was found between the size of NEBs (number of PNECs) and the number of BrdU-positive (divided) CLCs in the NEB ME following LPS challenge. The observed trend that larger NEBs harbored slightly more divided CLCs might simply be explained by the fact that the absolute number of CLCs is higher around large groups of PNECs.

In sham-treated mice (intratracheal instillation with saline), divided cells were also observed in an elevated percentage of NEBs (more than 23%), compared to untreated animals (less than 10%), but the total number of BrdU-positive cells was still limited compared to that observed following LPS challenge. Plethysmography showed that respiratory parameters did initially also change in sham-treated animals, but that they restored faster than in LPS-treated animals. Whereas BALF of untreated control mice harbored macrophages only, BALF of sham mice additionally contained neutrophils, but clearly less than BALF of LPS-instilled mice. Although the number of divided airway epithelial cells in sham-treated mice is small compared to that seen after LPS in our experiments, the preferential location of BrdU-labeled cells surrounding PNECs in the NEB ME is interestingly similar to the selective CLC proliferation seen after LPS treatment and suggestive of a joint mechanism of action, possibly involving early neutrophil mediators as discussed above.

Apart from CLCs (vCE cells), PNECs in the NEB ME have also been put forward as a cell type with potential airway epithelial stem cell characteristics [34, 35], although this hypothesis is still the subject of controversy.

The classic view that PNECs are terminally differentiated [76, 77], is challenged by the notion that repair of severe airway injury is associated with hyperplasia of PNECs [35]. Chemically or genetically induced full depletion of CCs revealed a typical proliferation of PNECs [36, 70]. Although at least subpopulations of PNEC-like progenitors are believed to give rise to CCs and even to alveolar epithelial cells during early development [30, 78, 79], most studies suggest that PNECs are not able to restore adult airway epithelium after ablation of all types of CCs [12]. Elimination of PNECs prior to CC depletion seems to have no apparent consequence for CC regeneration [34], but the same study reports that to some extent PNECs may contribute to CCs and ciliated cells following severe lung injury.

The presented data (LPS treatment; 48 h experimental window) show a very low number of divided PNECs in the NEB ME, which is not significantly different between LPS-challenged, sham and untreated control mice. In contrast to the well-illustrated proliferation of endocrine cells (PNECs) in addition to CLCs/vCE cells in several studies that are based on a full depletion of CCs [12, 35, 70], our LPS-based transient mild injury model for the selective proliferation of CLCs offers the important advantage that PNECs in the NEB ME are not affected by the procedure. The latter is in agreement with our observation that soluble mediators in BALF of LPS-challenged mice result in a calcium-mediated activation of CLCs but not of PNECs in the NEB ME in lung slices of control mice.

Certainly, PNECs can secrete regulatory factors –e.g. gastrin-releasing peptide (bombesin) and CGRP, potential epithelial cell mitogens [80]– that may support and regulate airway epithelial cell renewal/proliferation and differentiation. PNECs, however, are not only able to produce, store and secrete a variety of bioactive substances –some of which may directly influence CLCs [13]– but also to monitor calcium-mediated events in surrounding CLCs [14], and may therefore be involved in creating a niche to maintain the stem cell characteristics of CLCs.

## Conclusion

Based on a single low-dose intratracheal LPS instillation, a highly reproducible and minimally invasive lung inflammation model was generated and validated for inducing selective activation of a quiescent airway stem cell population –the so-called CLCs/vCE cells– in the NEB ME.

Important advantages compared to earlier models, which were mainly based on full ablation of CCs, are the absence of both severe epithelial injury and additional proliferation of endocrine cells (PNECs).

The fact that CLCs in the NEB ME can be activated from a silent to a dividing stem cell population in the absence of severe airway epithelial damage creates new opportunities for unraveling the cellular mechanisms/pathways regulating silencing, activation, proliferation and differentiation of this unique postnatal airway epithelial stem cell population.

The presented data are supportive of potentially important selective roles of the postnatal airway stem cell niche of the NEB ME, and enable the identification of pathways that should allow uncoupling of essential repair mechanisms from severe lung injury and inflammation.

## Abbreviations

[Ca^2+^]_i_: Intracellular calcium concentration
[K^+^]_o_: Extracellular potassium concentration
4-Di-2-ASP: 4-(4-diethylaminostyryl)-N-methylpyridinium iodide
BALF: Bronchoalveolar lavage fluid
BrdU: 5-bromo-2′-deoxyuridine
BSA: Bovine serum albumin
BW: Bodyweight
CAE: Control airway epithelium
CC: Clara cell
CCSP: Clara cell secretory protein
CGRP: Calcitonin gene-related peptide
CLC: Clara-like cell
DMEM-F-12: Dulbecco’s modified Eagle’s medium/F-12
EIP: End-inspiratory pause
GAD67: Glutamic acid decarboxylase 67
H&E: Hematoxylin and eosin
i.p.: Intraperitoneal
LCI: Live cell imaging
LMD: Laser microdissection
LPS: Lipopolysaccharide
Mc: Monoclonal
ME: Microenvironment
NEB ME: Neuroepithelial body microenvironment
NEB: Neuroepithelial body
PBS: Phosphate-buffered saline
Pc: Polyclonal
PD: Postnatal day
PF: Paraformaldehyde
PNEC: Pulmonary neuroendocrine cell
ROI: Region of interest
RT: Relaxation time
SCLC: Small cell lung carcinoma
SEM: Standard error of means
Te: Expiratory time
TV: Tidal volume
UP1: Urine protein 1
vCE: Variant CCSP-expressing
WT-Bl6: Wild type C57BL/6 J
